# *Strongyloides stercoralis* infection in marmosets: replication of complicated and uncomplicated human disease and parasite biology

**DOI:** 10.1186/s13071-014-0579-2

**Published:** 2014-12-12

**Authors:** Vitor Luís Tenório Mati, Pedro Raso, Alan Lane de Melo

**Affiliations:** Department of Parasitology, ICB, UFMG, Belo Horizonte, Brazil; Department of Pathological Anatomy and Legal Medicine, FM, UFMG, Belo Horizonte, Brazil

**Keywords:** Strongyloides stercoralis, Neotropical primate model, Experimental infection, Glucocorticoid, Hyperinfection, Disseminated infection

## Abstract

**Background:**

*Strongyloides stercoralis* can undergo an alternative autoinfective life cycle in the host, which, in some individuals can lead to a lethal infection. However, due to a number of factors, such as, the majority of those infected are from low-income backgrounds and the limitation in experimental models for studying human *S. stercoralis,* strongyloidiasis remains neglected. Improved knowledge of animal models that are susceptible to this parasite is needed in order to investigate the immunological mechanisms involved during infection and in particular to further understand the natural history of the autoinfective cycle.

**Methods:**

*Callithrix penicillata* were inoculated subcutaneously with 100 (n = 2), 300 (n = 4) or 500 (n = 9) third-stage infective larvae (L3i) of *S. stercoralis* of human origin. Three marmosets received smaller inocula (i.e., one received 100 and two received 300 L3i) to ensure a greater capacity to withstand the infection after immunosuppression, which was triggered by administration of dexamethasone during early patency. Qualitative faecal analyses began at 7 days post-infection (DPI), and semi-quantitative tests were also performed for the dexamethasone-treated primates and the three matched controls. During the necropsies, specimens of *S. stercoralis* were recovered and tissue fragments were processed for histopathology.

**Results:**

The mean prepatency and patency periods were 16.1 ± 3.0 and 161.1 ± 72.2 DPI, respectively. The marmosets typically tolerated the infection well, but immunosuppressed individuals exhibited higher numbers of larvae in the faeces and progressive clinical deterioration with late disseminated infection. In these cases, the number of females recovered was significantly higher than the number of inoculated L3i. Large quantities of larvae were observed migrating through the host tissues, and histopathology revealed pulmonary and intestinal injuries consistent with those observed in human strongyloidiasis.

**Conclusions:**

Both complicated and uncomplicated strongyloidiasis occur in *C. penicillata* that is described as a susceptible small non-human primate model for *S. stercoralis*. This host permits the maintenance of a human strain of the parasite in the laboratory and can be useful for experimental investigations of strongyloidiasis. In parallel, we discuss data related to the autoinfective cycle that provides new insights into the biology of *S. stercoralis*.

**Electronic supplementary material:**

The online version of this article (doi:10.1186/s13071-014-0579-2) contains supplementary material, which is available to authorized users.

## Background

*Strongyloides stercoralis* (Bavay, 1876), which is the main aetiological agent of human strongyloidiasis, is the only medically important nematode that can multiply in the host via an autoinfective cycle to reach critical levels and cause death [1]-[3]. A number of factors, such as the major involvement of low-income individuals and residents of developing countries [1],[4],[5], limitations for proper diagnosis (e.g., up to 70% of cases are not detected by conventional methods [6]) and the absence of viable models for the study of *S. stercoralis* of human origin, have contributed to making strongyloidiasis a neglected disease; although strongyloidiasis has been known since the nineteenth century and has a wide geographical distribution, this disease remains neglected, with estimates indicating that this infection currently affects at least 370 million people around the world [4]. However, a recent meta-analysis conducted using data from several countries indicates that even this number may be underestimated [5]; this study reported that in the People’s Republic of China alone, the average prevalence of strongyloidiasis might reach 14% (i.e., approximately 200 million cases).

The relative lack of interest in this infection among the research community has generated discussion as to whether strongyloidiasis is the most neglected of the neglected tropical diseases (NTDs) [2]. Even the World Health Organization (WHO) failed to include strongyloidiasis in its original list of 17 NTDs, although this helminthiasis is now one of the seven “other neglected conditions” together with chronic suppurative otitis media, scabies, snakebites, mycetoma, nodding syndrome and podoconiosis [7]. Concern about strongyloidiasis has augmented, possibly due to the increasing number of fatal cases, including patients who die due to iatrogenic causes related to the inappropriate use of immunosuppressive drugs [8]-[11]. Complicated strongyloidiasis (i.e., hyperinfection and/or dissemination of *S. stercoralis*) often occurs during host immunosuppression, particularly during prolonged high-dose corticosteroid treatment, which is the main risk factor for disseminated disease [12],[13]. In a recent review of clinical cases of complicated strongyloidiasis, 67% (164/244) of patients had received corticosteroids [11].

The lack of available direct experimental evidence has led to the discussion of interesting immunological factors involved in *S. stercoralis* dissemination during human strongyloidiasis in patients with immunosuppression caused by treatment with corticosteroids or by infection with the Human Immunodeficiency Virus (HIV) or Human T-Lymphotropic Virus type 1 (HTLV-1). The immunosuppression induced by viruses and by the administration of this class of drug is similar because the immunological activity of the organism is affected primarily by interference with cell-mediated immunity [14]-[16]. Differences in the immunology of the HIV and HTLV-1 viruses in the host explain the increased number of cases of disseminated strongyloidiasis in patients with prior HTLV-1 infection. HIV infection usually results in reduced T helper cell type 1 (Th1) responses, while T helper cell type 2 (Th2) responses are unaffected or even increased [17]. In contrast, individuals infected with HTLV-1 exhibit a predominantly Th1 response [17],[18]. During human co-infection with *S. stercoralis* and HTLV-1, increased production of interferon-γ (IFN-γ) and interleukin-10 (IL-10) is associated with decreased production of interleukin 5 (IL-5) by peripheral blood mononuclear cells (PBMCs). In addition, reduced levels of immunoglobulin E (IgE) are observed. These findings indicate a change from a Th2 to Th1 response [19]. The modulation of the immune response that is induced by IFN-γ produced by activated T cells in patients harbouring HTLV-1 has been suggested to cause reduced serum IgE levels [20]. PBMCs from individuals infected with both *S. stercoralis* and HTLV-1 also exhibit reduced production of interleukin-4 (IL-4). Even after elimination of *S. stercoralis* by ivermectin treatment, lower blood IgE levels and eosinophil counts were observed in these patients than in patients infected with only *S. stercoralis*[21]. Investigations performed in both humans and experimental models with different species of intestinal nematodes demonstrated that the production of immunoglobulins and Th2 cytokines is beneficial to the host. IL-4 and IL-5 stimulate the migration of eosinophils and IgE production, which play a central role in mast cell degranulation, preventing parasite attachment and invasion of the host intestinal mucosa. In addition, the increased frequency and intensity of peristaltic waves can contribute to the expulsion of intestinal nematodes [9],[22],[23]. Eosinophils and antibodies participate in human immunity against *S. stercoralis* larvae and are likely to reduce the risk of developing severe forms of the disease because immunoglobulin levels and eosinophil numbers were significantly lower in patients with complicated disease than in patients with uncomplicated disease [24]. Additionally, the occurrence of both humoral and cellular responses, with increases in mast cell numbers and histamine levels in the host intestinal mucosa, was previously demonstrated in patas monkeys (*Erythrocebus patas*) infected with a human strain of *S. stercoralis*[25]-[27]. The mechanisms related to *S. stercoralis* dissemination in patients receiving corticosteroids are not yet fully known. Among the various effects induced by corticosteroid treatment during different pathological processes, reduced expression of pro-inflammatory cytokines, chemokines, adhesion molecules and enzymes related to inflammation has been reported [14],[28]. These drugs also affect the number and characteristics of circulating cells in the vertebrate host and the migration of leukocytes to the site of infection [29]-[31]. The inhibition of cellular proliferation and the induction of apoptosis in immune cells, including lymphocytes and eosinophils, can also occur [14],[30],[32]. Furthermore, although the effects of corticosteroids on the host immune system are significant, some evidence suggests that the Th2 response may be more affected by treatment than the Th1 response; in these cases, a consequent reduction in the number and activity of eosinophils and mast cells is related to the dissemination of *S. stercoralis*[9],[27],[33]. Moreover, an alternative hypothesis has been proposed to explain the occurrence of complicated strongyloidiasis. Although this hypothesis is not yet experimentally confirmed, it suggests that endogenous or exogenous corticosteroids may share structural similarities with nematode ecdysteroids. As a result, corticosteroids may favour molting of rhabditiform larvae, resulting in a substantial increase in the number of infective larvae of *S. stercoralis* that have the ability to penetrate the host intestinal mucosa (i.e., auto-infection) [34].

Experimental studies of *S. stercoralis*, particularly those related to complicated strongyloidiasis, are needed to obtain a better understanding of this infection and facilitate the development of new strategies for the prevention and treatment of human disease. Even aspects of the basic biology of *S. stercoralis*, such as the replication, development and migration of this pathogen in the host, requires further investigation. Mice typically do not support the development of *S. stercoralis*[35]. The use of jirds (*Meriones unguiculatus*) to study the parasite has limitations, such as the lack of available immunological information for these rodents and the small number of larvae that are released in the faeces of these rodents, which makes it difficult to maintain *S. stercoralis* in the laboratory [36]. The canine model, which was used in early experimental studies of strongyloidiasis [37],[38], was popular in the 1980s and 1990s and provided interesting results from experiments using nematode strains isolated from dogs [39]-[45] and humans [46]-[49]. However, when dogs were experimentally infected with human isolates of *S. stercoralis*, a reduction in the prepatency of infection and an abolition of the hyperinfection process and any type of extra-intestinal migration of the parasite, including during immunosuppression, were observed after a small number of passages in the new host [49]. The extremely variable susceptibility of dogs to *Strongyloides* spp*.*[37],[38],[50],[51] is considered the main drawback of the canine model, but the large phylogenetic distance between dogs and humans, practical difficulties related to the size of these animals and high housing and maintenance costs have also restricted the use of this model [46].

A non-human primate model is desirable because, in addition to the phylogenetic proximity and physiological similarities between humans and other primates, strongyloidiasis cases with clinical and pathological features similar to those observed in human disease, including fatalities, have been reported in gibbons [52], orangutans [53], gorillas and chimpanzees [54]. Captive patas monkeys also presented fatal strongyloidiasis [55], and this observation stimulated the previously described studies [25]-[27] using a human isolate of *S. stercoralis* in this primate; however, the large size of this potential model and practical difficulties impede wider use of this model. The use of marmosets as a nonhuman primate model is a viable alternative due to the small size and easy handling of these Neotropical primates. Indeed, we have already suggested *Callithrix penicillata* as a model for the study of strongyloidiasis [56], but the infecting parasite species was *Strongyloides venezuelensis*.

The infection of marmosets with human *S. stercoralis* was standardized in this study, and the natural history of complicated (i.e., after immunosuppression using dexamethasone) and uncomplicated strongyloidiasis are reported. The data obtained illustrate that the use of marmosets as a model for the study of strongyloidiasis is promising, including for studies of various aspects of disseminated infection after the administration of corticosteroids. Additionally, new considerations related to the biology of the nematode, particularly with respect to its development and migration within the host, are discussed.

## Methods

### Nonhuman primates

Fifteen adult male specimens of captive-born *C. penicillata* that weighed 370 ± 45 g and were reared in the marmoset colony in the Instituto de Ciências Biológicas (ICB) at the Universidade Federal de Minas Gerais (UFMG) were selected for experimental study. Briefly, in this colony, small families of marmosets are kept in different rooms (1 m wide, 3 m long, and 2.5 m high) for breeding. These nurseries have brick walls, a roofed section and another section bounded by an iron grid at the top to allow direct exposure to sunlight. The marmosets do not have eye contact with the adjacent room. Food is distributed twice per day: fruit in the morning and a feed ration *ad libitum* in the afternoon [57]. When necessary the morning feed is replaced by egg yolks or gelatin containing a multivitamin supplement [58].

The specimens used in the present study were placed in individual cages that each had a PVC tube suspended near the top, providing a nest for the marmosets to rest and hide. The animals remained housed in the cages throughout the period when the experiments were conducted, and the clinical state of the animals was checked every day.

### Considerations for experimental design

This study included two experiments related to strongyloidiasis in *C. penicillata*. Experiment I aimed to accomplish the standardization of primary infection. All marmosets that were experimentally infected with *S. stercoralis* (n =15) were considered in this study. Experiment II aimed to assess the effect of immunosuppression induced by corticosteroids on experimental infection. Among the 15 animals infected with the parasite, three received immunosuppressive therapy using dexamethasone and three others were matched as untreated controls (see below).

### Parasites

The strain of *S. stercoralis* used in this study was isolated from human faecal material from individuals living in the state of Minas Gerais, Brazil. The strain was maintained in the laboratory by means of successive passages in marmosets.

### Obtaining and counting the infective larvae

To obtain infective third-stage filariform larvae (L3i), faecal cultures were set up. Faeces from humans or marmosets were mixed with vermiculite and then incubated in a chamber heated to 27°C for a period of between 48 and 72 hours. Recovery of the larval forms was made using the Baermann method, as modified by Moraes [59], and the suspension containing L3i were decontaminated according to Martins *et al.*[60]. Aliquots of 25 μl were collected with the aid of an automated pipette and were placed as drops on slides for counting to be performed. The mean number was calculated from evaluations on at least three aliquots, and the suspension was then diluted or concentrated in order to attain a final concentration of 100, 300 or 500 L3i in 0.5 ml of distilled water.

### Infection of the marmosets

Each marmoset was infected by means of subcutaneous inoculation of 0.5 ml of a suspension containing L3i of *S. stercoralis* obtained from human (H) or marmoset (M) faecal cultures. Animals P1 and P2 (n = 2) and P3 and P4 (n = 2) were inoculated with 100 and 500 L3i/H, respectively. The other marmosets received 100 (i.e., P5 and P6; n = 2), 300 (i.e., P7 and P8; n = 2) and 500 (i.e., P9, P10, P11, P12, P13, P14 and P15; n = 7) L3i/M.

### Dexamethasone administration

Three primates (i.e., P2, P6 and P8) received short-term treatment with 2.5 mg/kg of dexamethasone (Decadron**®**) subcutaneously for five consecutive days, starting the 4^th^ (P6 and P8) or 5^th^ (P2) week post-infection [56]. This drug is a glucocorticoid with a known immunosuppressive effect. Treated and control primates were matched according to weight and the source and number of inoculated L3i. These animals received a lower number of L3i (i.e., 100 or 300) to ensure that they could tolerate the infection and to model the potential for complicated disease for a longer period of time after immunosuppression.

### Coproparasitological examination

Beginning one week after infection, fresh samples of faeces from all of the marmosets were collected 5 days per week throughout the period of the experiment in order to make qualitative analyses using the spontaneous sedimentation [61] and modified Baermann [59] methods. Approximately 2 g of stool was processed for the first technique, and faecal cultures were started with the remainder of the samples for use in the latter method. For the spontaneous sedimentation technique, 3 slides were analyzed per day and all of the material obtained using the Baermann-Moraes method was examined. Examinations of each animal’s faecal samples were performed until parasitological cure (i.e., after at least 12 consecutive weeks in which the samples exhibited negative faecal results).

For the marmosets used in experiment II, a semi-quantitative estimation of the number of nematode forms in the immunosuppressed and control groups was conducted during spontaneous sedimentation assays [62] that were performed until 10 weeks of infection. Because the amount of faeces used in these assays and the volume analyzed on each slide were approximately constant, positive results were categorized by convention as + (< 10 L1 per slide), ++ (10 to 100 L1 per slide) and +++ (> 100 L1 per slide) by the same author.

### Necropsy, recovery of *S. stercoralis* and histopathology

The primates that spontaneously died and the marmoset P4 that was euthanized were necropsied. The intestines of these animals were opened longitudinally in Petri dishes containing a 0.85% solution of sodium chloride. After scraping the intestines using a glass slide, the presence of larvae and parasitic females of *S. stercoralis* in the host tissues was directly evaluated using a stereomicroscope. The samples were transferred to a sieve that was placed on sedimentation glass containing saline and incubated at 37°C for three hours. Nematodes were recovered from the sediment, counted and fixed in 10% formalin. We also searched for specimens of *S. stercoralis* in other organs, including the lungs, liver, spleen and kidneys.

Parasitic females were classified according to the criteria described by Faust [63]. The juvenile female has a vulva that is already evident, but its total length does not exceed 1.5 mm, which is shorter than the total length of adult females. The ratio “esophagus length/total length” (approximately 1/3) is intermediate between the ratios observed in L3i (1/2) and adult females (1/4), while the tail (i.e., the distance between the anus and the posterior end) is proportionally longer in juvenile females.

Small fragments of intestines and other organs were fixed in 10% neutral buffered formalin, and the tissues were subsequently paraffin-embedded, cut into 4-μm sections and stained with hematoxylin-eosin. Images of the histopathological slides were captured using a Leica DM500 microscope (Leica Microsystems, Heerbrugg, St. Gallen, Switzerland) with a video camera attached to a PC.

### Statistical analysis

The Shapiro-Wilk test was used to determine normality. Data was analysed using a Student’s t-test when the distribution was Gaussian and the Kruskal-Wallis test was used when the distribution was non-parametric.

### Ethics

The use of marmosets in this biomedical research study was authorized by the Instituto Brasileiro do Meio Ambiente e Recursos Naturais Renováveis (IBAMA), and the experimental procedures were conducted in accordance with the precepts of the local animal research ethics committee of UFMG (CETEA/CEUA) (registration number of the ethical authorization 167/06, renewed in 2012).

## Results

### The marmoset model is susceptible to a human isolate of *Strongyloides stercoralis*

**Figure 1:**
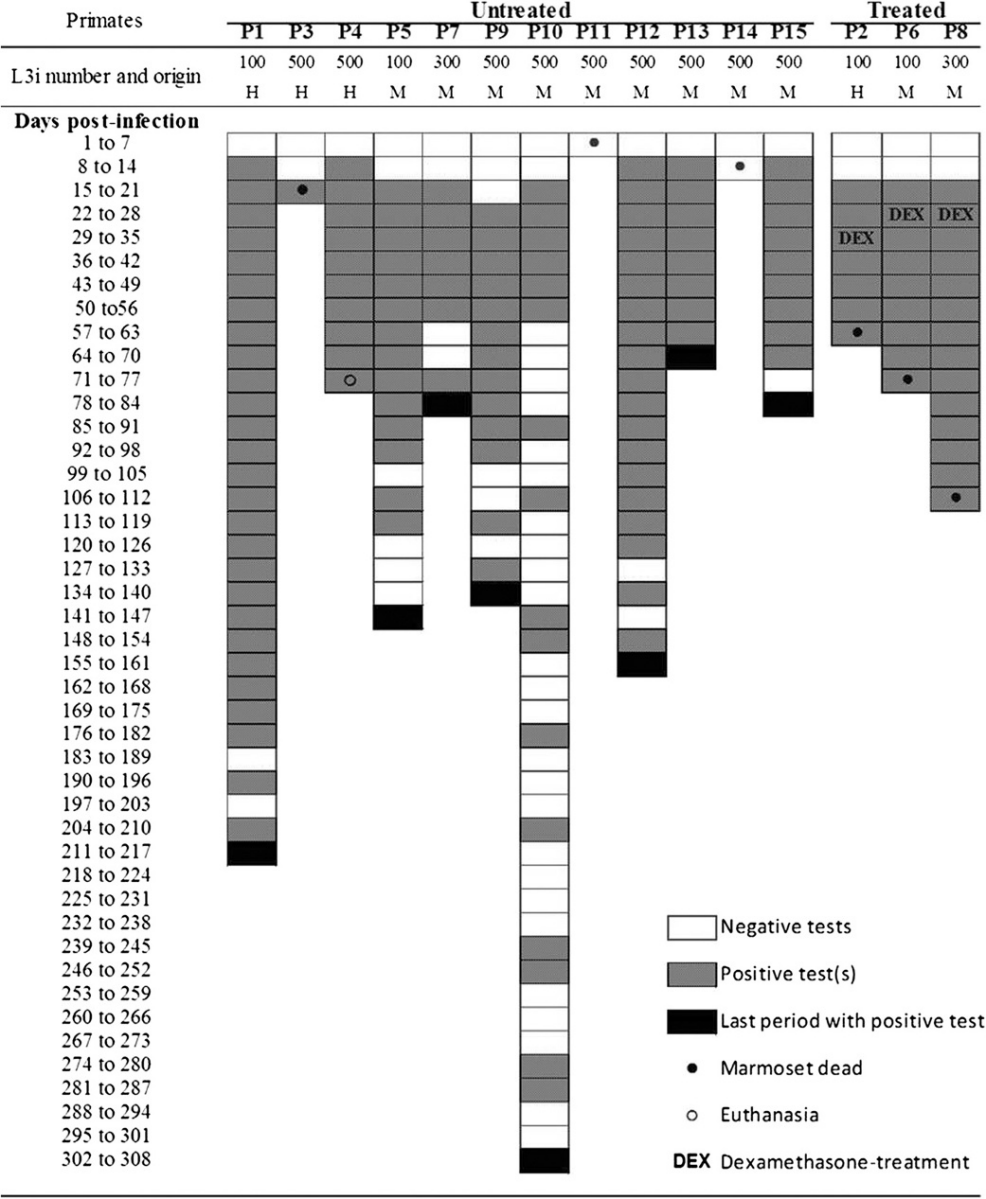
**Results of faecal analyses of** ***Callithrix penicillata*** **during experimental infection with** ***Strongyloides stercoralis*** **.** Each week, faecal material from the marmosets that corresponded to at least five different days was evaluated and analyses were performed until confirmation of parasitological cure or death of the primate. L3i = inoculated infective larvae, H = human, M = marmoset, DEX = dexamethasone treatment.

Marmosets were susceptible to *S. stercoralis*, irrespective of the number (i.e., 100, 300 or 500) and origin (i.e., human or marmoset) of inoculated L3i. The infection of *C. penicillata* was confirmed by the presence of the parasite in faeces and/or in necropsies (Tables 1, 2; Figure 1). Most infected marmosets (86.7%, 13/15) released rhabditiform larvae of *S. stercoralis* in their faeces. Marmosets P11 and P14, who died before the patent period, were exceptions. However, in these animals, specimens of *S. stercoralis* and/or tissue lesions induced by the parasite were observed.

**Table 1:** **Percentage of parasitic females recovered after inoculation of infective larvae of** ***Strongyloides stercoralis*** **into** ***Callithrix penicillata***

	Primates	Days post-infection	% of infective larvae of***S. stercoralis***recovered as parasitic females			
			Small intestine	Large intestine	Other organs	Total
**Untreated** (uncomplicated strongyloidiasis)	**P3**	21	21.6 (108/500)	2.2 (11/500)	0	23.8 (119/500)
	**P4**	73	15.8 (79/500)	1.6 (8/500)	0	17.4 (87/500)
	**P14**	12	10.6 (53/500)	0 (0/500)	0	10.6 (53/500)
**Mean ± sd**			16.0 ± 5.5	1.3 ± 1.1	0	17.3 ± 6.6
**Treated with dexamethasone** (complicated strongyloidiasis)	**P2**	60	719.0 (719/100)	18.0 (18/100)	10.0 (10/100)	747.0 (747/100)
	**P6**	71	613.0 (613/100)	37.0 (37/100)	7.0 (7/100)	657.0 (657/100)
	**P8**	109	156.0 (468/300)	3.0 (9/300)	11.7 (35/300)	170.7 (512/300)
**Mean ± sd**			496.0 ± 299.2	19.3 ± 17.0	9.6 ± 2.4	524.9 ± 310.5
**p - value**			< 0.05		< 0.005	< 0.05

**Table 2:** **Results of a semi-quantitative faecal analysis in immunocompetent and dexamethasone-immunosuppressed marmosets during experimental strongyloidiasis**

Primates		Untreated (uncomplicated strongyloidiasis)			Treated with dexamethasone (complicated strongyloidiasis)		
		P1	P5	P7	P2	P6	P8
**L3i**	Number	100	100	300	100	100	300
	Source	H	M	M	H	M	M
**Days post-infection**							
1 to 7		-	-	-	-	-	-
8 to 14		+	-	-	-	-	-
15 to 21		+	+	+	+	+*	+*
22 to 28		+ +	+	+ +	+ +*	+	+
29 to 35		+ +	+ +	+	+ +	+ +	+ + +
36 to 42		+	+ +	+	+	+ +	+ + +
43 to 49		+	+	++	+ + +	+ + +	+ +
50 to 56		+	+	+	+ + +	+ +	+ +
57 to 63		+	+	-	+ + +	+ + +	+ + +
64 to 70		+	+	-		+ +	+ + +
71 to 77		+	+	+		+ + +	+ +
78 to 84		+	+	+			+ +
85 to 91		+	+	-			+ +
92 to 98		+	+	-			+
99 to 105		+	-	-			+**

L3i = inoculated infective larvae.

H = human.

M = marmoset.

+, ++, +++ indicate low, moderate, and high numbers of larvae in the faeces, respectively, and - indicates a negative result.

*Course of dexamethasone treatment.

**Dead at 109 days post-infection.

No significant differences in the natural course of infection were observed among immunocompetent primates infected with different numbers of L3i, except for some deaths that occurred during early infection in marmosets infected with 500 L3i (see subtopic on lethality). Moreover, the origin of the L3i (i.e., from human or marmoset faecal cultures) also failed to influence the evaluated parameters. The mean prepatent and patent periods were 16.1 ± 3.0 and 161.1 ± 72.2 days post-infection (DPI), respectively. Rhabditiform larvae of *S. stercoralis* were first observed during faecal analysis of marmosets at a minimum of 13 (P4 and P13) and a maximum of 22 DPI (P9). Early (P13) and late (P10) parasitological cures were observed at 70 and 308 DPI, respectively (Figure 1).

### Reproducibility of the clinical forms of human strongyloidiasis in immunocompetent and dexamethasone-immunosuppressed experimental hosts

Strongyloidiasis in marmosets was representative of observations of human infection with *S. stercoralis*. Based on the evaluation of parasitological and anatomo-clinical parameters, the two forms of human disease that occur in the presence and absence of immunosuppression were also observed in experimentally infected marmosets.

I. Uncomplicated strongyloidiasis

In the absence of exogenous immunosuppression induced by dexamethasone administration, most specimens of *C. penicillata* tolerated the infection with *S. stercoralis* well, particularly after established patency. In addition to supporting the complete development of the parasite, the immunocompetent marmosets exhibited signs of intestinal strongyloidiasis, with outcomes representative of those observed in immunocompetent humans infected with the nematode.

II. Complicated strongyloidiasis

The animals inoculated with dexamethasone exhibited reversible adverse signs related to treatment with glucocorticoids, such as oedema and skin changes, indicating immunosuppression during and after the administration of the drug. However, the main changes in the natural course of infection were observed later, culminating in the typical scenario of hyperinfection followed by dissemination of *S. stercoralis* and death of the host.

### Characteristics of complicated and uncomplicated strongyloidiasis in marmosets

#### Parasitological faeces analysis

An alternation between positive and negative results was observed during uncomplicated infection in primates. Long periods of serial positive tests and serial negative tests were observed. The largest continuous periods of positive (25 weeks) and negative (four weeks) results were observed in marmosets P1 and P10, respectively. During the patent period, negative faecal analyses became proportionately more common for most untreated animals as the duration of infection progressed (Figure 1). However, the group that received dexamethasone exhibited no negative parasitological results during the patent period of the infection and a greater number of larvae of *S. stercoralis* was observed in the stools of these animals than in the stools of the animals in the control group (Table 2). Slides of faecal material from immunosuppressed marmosets showed full fields of parasite larvae under light microscopy, particularly in the last weeks before the death of each animal. In the days preceding the death of the animal P8, faecal analyses were impossible due to a lack of evacuation.

#### Lethality

The overall lethality rate observed among the marmosets in the first weeks of experimental strongyloidiasis, prior to treatment of primates with dexamethasone, was 20% (3/15). The proportion of deaths was affected by the number of inoculated L3i. All deaths of untreated marmosets occurred early in infection in animals that received 500 L3i parasites (at 3, 12 and 21 DPI for P11, P14 and P3, respectively). Moreover, when considering only this initial period prior to the third week after L3i inoculation, the lethality among marmosets infected with 500 L3i of *S. stercoralis* was 33.3% (3/9) and the lethality was 0 (0/6; also including the three animals that were subsequently immunosuppressed) among those inoculated with 100 or 300 L3i.

In experiment II, the lethality of *S. stercoralis* infection in dexamethasone-treated marmosets was 100% (3/3) and the lethality was zero (0/3) in the respective controls (Figure 1, Table 2). It is worth noting that unlike the deaths of untreated animals, the primates who received the immunosuppressive regimen and developed complicated strongyloidiasis died later in infection, between 4 and 12 weeks after the administration of the drug.

In total, 7 necropsies were performed, corresponding to the 6 primates who died spontaneously due to strongyloidiasis and the euthanized animal P4.

#### Clinical and pathological aspects

The mean weight of the primates with uncomplicated disease did not vary significantly over the natural course of strongyloidiasis. However, in marmosets with the complicated form of infection, a reduction of adipose tissue and an average loss of weight of 28% (p < 0.05) occurred gradually and steadily until the time of death. Although most immunocompetent primates infected with *S. stercoralis* appeared healthy during the experiment, signs such as bristling fur, respiratory effort/dyspnea and appetite loss were observed, particularly early in the infection (i.e., during the pulmonary phase of larval migration). Subsequently, gastrointestinal manifestations emerged; these manifestations included diarrhea, which was sometimes intermittent and alternated with periods of constipation. A gradual clinical improvement then occurred in the animals that healed spontaneously, despite varying temporal evolution. The marmosets that received dexamethasone exhibited more severe clinical manifestations due to hyperinfection and dissemination of the parasites. Prior to the deaths of these animals, in addition to intestinal manifestations, the late involvement of the respiratory system was observed. In particular, in marmoset P2, the nervous system was also affected.

**Figure 2:**
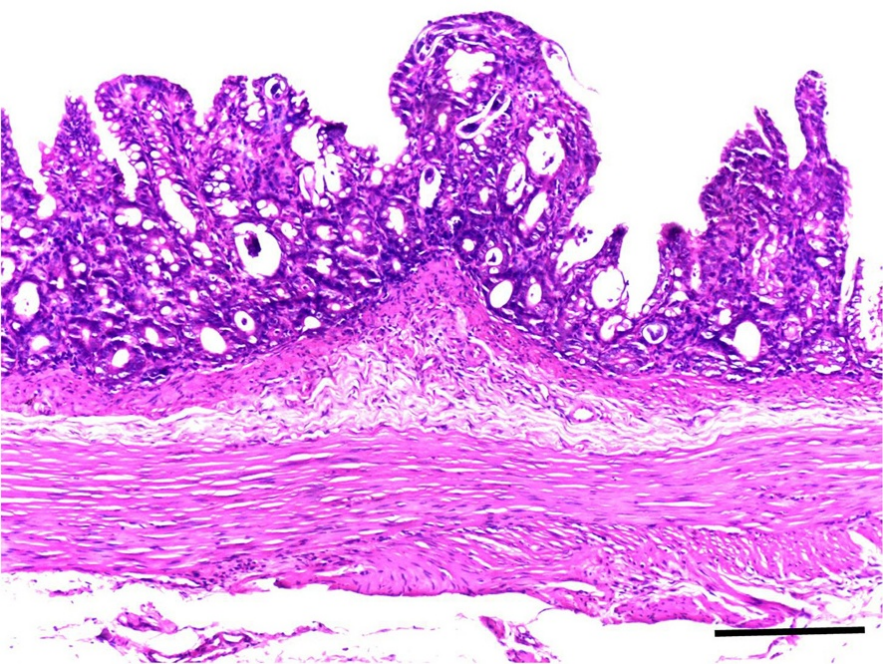
**Histological section of the small intestine of** ***Callithrix penicillata*** **experimentally infected with** ***Strongyloides stercoralis*** **.** Note the parasitic elements in the mucosa and the detachment of villi. HE stain. Bar = 200 micrometers.

Histopathology revealed sections of adults and larvae of *S. stercoralis* in the intestinal mucosa of primates (Figure 2). Similar to the semiologic and lethality findings, the pathology also varied depending on the presence or absence of prior immunosuppressive treatment with dexamethasone. The organs involved and the characteristics and intensity of the microscopic lesions were dependent on the time elapsed from infection to necropsy.

Lung injuries in untreated *C. penicillata,* such as interstitial pneumonitis, hyperemia, oedema, thromboembolism and alveolar hemorrhage, were observed during periods corresponding to the primary pulmonary migration of *S. stercoralis*, and basophilic structures were observed in the alveolar lumen in marmoset P11, which died at 3 DPI. In the small intestine, the presence of an inflammatory infiltrate consisting primarily of mononuclear cells was observed in the lamina propria. In restricted areas, involvement of the submucosa, with changes in the structure of the villi, particularly atrophy, was observed during uncomplicated strongyloidiasis. However, in animals with *S. stercoralis* dissemination, pulmonary lesions similar to those described above were observed, but these lesions had a late onset and occurred to a greater extent. Furthermore, severe enteritis, with a mononuclear cell inflammatory infiltrate that was mixed with polymorphonuclear cells in some areas and reached the deeper layers of the organ, was observed. Areas of complete destruction of villi, with erosions and ulcerations, hyperemia and oedema of the submucosa and subserosa were observed. Odematous colitis related to larval migration was another common finding.

#### Recovery of larvae and parasitic females of *S. stercoralis*

In all necropsied marmosets, excluding the primate that died at 3 DPI, parasitic females of *S. stercoralis* were found, primarily in the host small intestine. The mean percentage recovery of parasitic females from untreated primates was 17.3 ± 6.6% of the total inoculated L3i. In dexamethasone-treated marmosets, this value reached 524.9 ± 310.5% (p <0.05) and was significantly higher. Surprisingly, the number of recovered *S. stercoralis* females was more than 7 times the L3i inoculum in marmoset P2 (Table 1). The immunosuppressed marmosets whose deaths were related to the dissemination of the nematode harboured similar total numbers of parasitic females (512, 657 and 747 in P8, P6 and P2, respectively) regardless of the number of larvae inoculated (i.e., 100 or 300 L3i). An average of 638.7 ± 118.6 parasitic females were recovered from these 3 primates; in contrast, this value was much lower (86.3 ± 33) among primates who did not receive dexamethasone.

**Figure 3:**
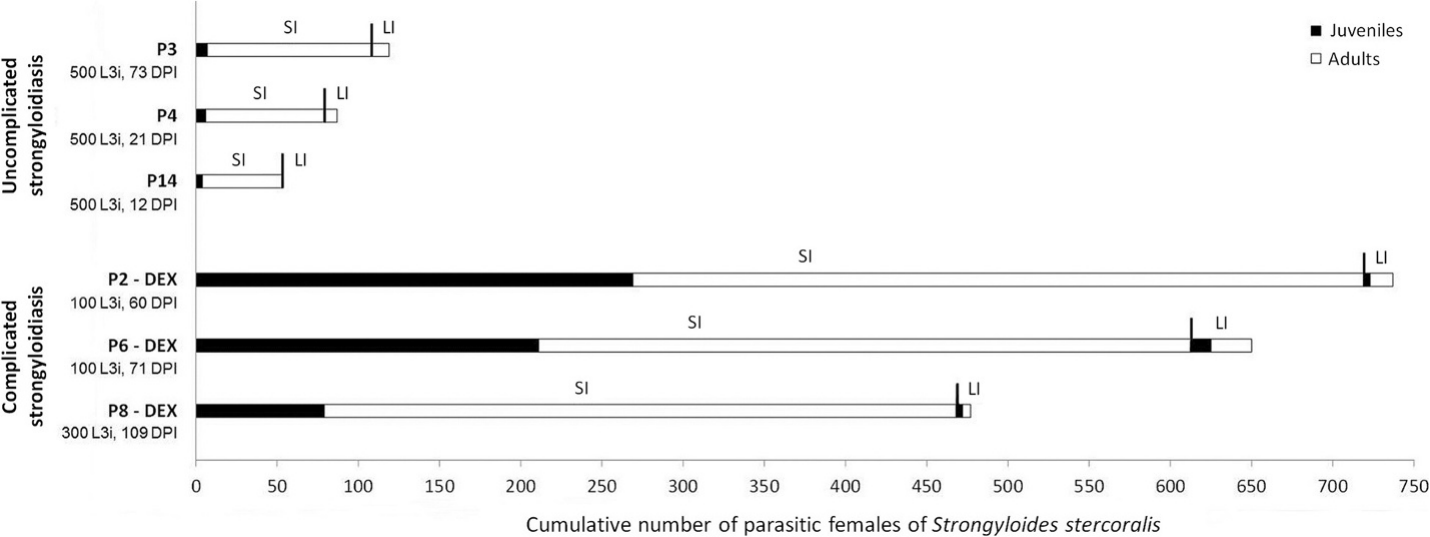
**Cumulative number of parasitic females of** ***Strongyloides stercoralis*** **recovered from the small and large intestines of** ***Callithrix penicillata*** **according to the degree of development (i.e., juvenile or adult) and the location.** SI = small intestine, LI = large intestine, DEX = previous treatment with dexamethasone, L3i = inoculated infective larvae; DPI = days post-infection.

The auto-infection indeed occurred in immunocompetent and immunosuppressed marmosets and juvenile females of *S. stercoralis* were observed in their intestines, even during more advanced infection periods. The proportion of juvenile parasites in the small intestine in untreated marmosets was small, but the absolute number was similar to the mean quantity of parasitic females observed in the large intestines. This number is proportionally higher than the number of *S. stercoralis* females observed in the same organ in marmosets with complicated disease. However, the number of *S. stercoralis* parasitic females in the large intestine of marmosets that exhibited a severe form of infection failed to increase with the same ratio (Table 1, Figure 3).

**Figure 4:**
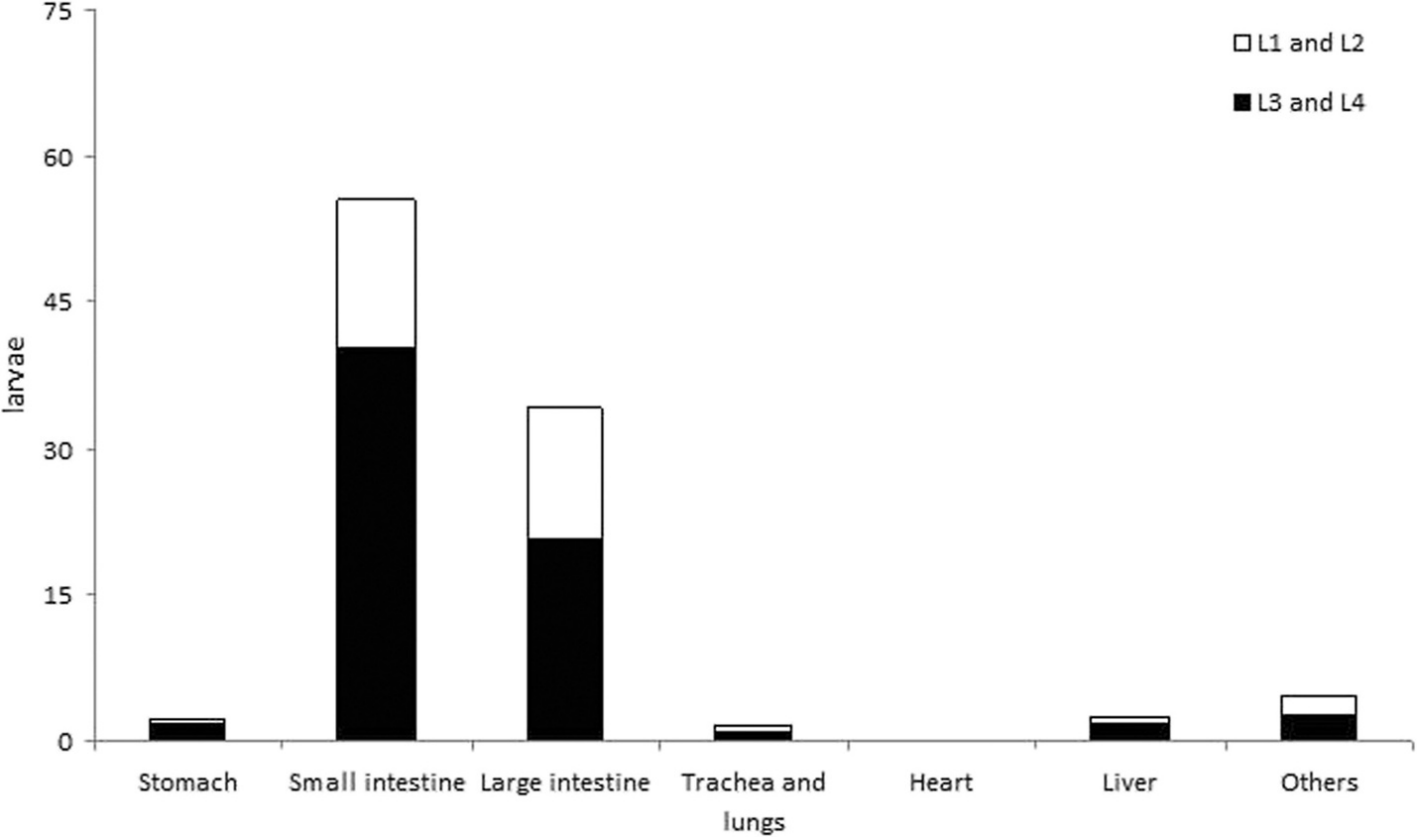
**Percentage of** ***Strongyloides stercoralis*** **larvae recovered from different organs in three specimens of** ***Callithrix penicillata*** **that died due to parasite dissemination after dexamethasone treatment.** L1 and L2 = rhabditiform larvae of the first and second stages; L3 and L4 = filariform larvae of the third and fourth stages.

During necropsies of immunocompetent *C. penicillata*, a small number of filariform larvae of *S. stercoralis* were found migrating in the intestines and more rarely in the lungs (i.e., one larva in one animal). Most of the larvae observed in these primates were rhabditiform and were primarily found free in the gut lumen. Conversely, in all immunosuppressed marmosets, variable forms of *S. stercoralis* (i.e., mostly rhabditiform and infective larvae but also juvenile and adult parasitic females) were recovered from the intestines and from extraintestinal sites such as the stomach, trachea and lung, liver, gallbladder and extrahepatic biliary tract, heart, kidney and spleen (Table 3). A total of 4,652 specimens of *S. stercoralis* of different developmental stages were analyzed, and the mean percentages of rhabditiform larvae, filariform larvae, juvenile females and adult females were 19.7%, 39.1%, 13.1% and 28.1%, respectively. Even in cases of disseminated infection, the intestines contained the greatest number of *S. stercoralis* larvae and females among the evaluated organs. Considering the distribution of only larval forms among these same organs, a small percentage of parasites was found in the trachea and lungs, with a similar value to that observed in the liver (Figure 4). The muscles and the skin were not routinely assessed, however, approximately 500 larvae were found in a fragment of skin and appendages (approximately 1.5 cm^2^) from a specimen with eczema (P8), this is of interest.

**Table 3:** **Number of larvae and females of** ***Strongyloides stercoralis*** **recovered from different organs in three specimens of** ***Callithrix penicillata*** **that died due to parasite dissemination after dexamethasone treatment**

Primates	Developmental stage	Organs							
		Stomach	Small intestine	Large intestine	Trachea and lungs	Heart	Liver, gall-bladder and biliary ducts	Others*	Total
P2	L1 and L2	0	94	84	5	1	6	7	197
	L3 and L4	5	415	230	7	1	18	20	696
	Juvenile ♀	2	269	4	1	0	1	2	279
	Adult ♀	0	450	14	2	0	1	1	468
	Total	7	1,228	332	15	2	26	30	1,640
P6	L1 and L2	2	99	63	2	0	2	12	178
	L3 and L4	17	197	106	4	0	9	11	344
	Juvenile ♀	2	211	12	1	0	1	0	227
	Adult ♀	2	402	25	1	0	0	0	430
	Total	23	909	206	8	0	12	21	1,179
P8	L1 and L2	16	217	245	14	0	9	40	541
	L3 and L4	15	478	212	10	0	20	45	780
	Juvenile ♀	7	79	4	2	0	7	6	105
	Adult ♀	8	389	5	3	0	0	2	407
	Total	46	1,163	466	29	0	36	93	1,833
Mean	L1 and L2	6.0	136.7	130.7	7.0	0.3	5.7	19.0	305.3
	L3 and L4	12.3	363.3	182.7	7.0	0.3	15.7	25.3	606.7
	Juvenile ♀	3.7	186.3	6.7	1.3	0	3.0	2.7	203.7
	Adult ♀	3.3	413.7	14.7	2.0	0	0.3	1.0	435.0
	Total	25.3	1,100.0	334.8	17.3	0.6	24.7	48.0	1,550.7

*Pelvic and other abdominal tissues and organs.

L1 and L2 = rhabditiform larvae of the first and second stages.

L3 and L4 = filariform larvae of the third and fourth stages.

♀ = parasitic female.

## Discussion

The inability to maintain *S. stercoralis* of human origin in the laboratory and the nearly complete absence of viable models for the study of auto-infection, hyperinfection and dissemination of this parasite have hampered our understanding of various aspects related to the biology and pathophysiology of this parasite. Marmosets are susceptible to infections with a number of parasites of medical importance [64]-[70], and the use of these animals has advantages over other non-human primates: smaller size (i.e., weight of only approximately 400 g), greater abundance of some species in nature, ease of captive breeding and handling, reduced maintenance costs and a relatively lower risk for the transmission of zoonoses to humans [56],[64],[71],[72]. In this study, specimens of *C. penicillata* were susceptible to infection with *S. stercoralis* and complicated and uncomplicated disease occurred, confirming that these primates are a promising model for studying strongyloidiasis [56].

The infection that occurred in dexamethasone-untreated marmosets was representative of the intestinal strongyloidiasis that occurs in immunocompetent patients, in which a balance in the host-parasite relationship occurs, with absent or minimal auto-infection. Immunosuppressed primates exhibited changes in the host-parasite relationship that resulted in *S. stercoralis* dissemination, as observed in humans with immune deficits. The signs corresponding to both of the forms of strongyloidiasis that were observed in *C. penicillata* are consistent with valuable information related to human infection. In humans there is a broad spectrum of clinical symptoms and infected individuals can be asymptomatic or present severe and fatal forms of the disease as a result of *S. stercoralis* dissemination, which is commonly observed when host immunity is impaired [1],[2],[8],[73]. Although most animals resisted experimental strongyloidiasis, during early infection, some marmosets inoculated with a higher number of L3i died. This point of infection was critical; in addition to these deaths, more significant clinical manifestations were observed. Over time, the immunocompetent marmosets began to better tolerate the infection, and clinical status improved, with no new deaths. This finding suggests that a more balanced host-parasite relationship occurs as the infection becomes chronic, perhaps due to host immunomodulation. However, when dexamethasone was administered, fatal cases occurred in all marmosets that had been infected with fewer L3i than the respective controls, which progressed to spontaneous healing.

The current understanding that a relationship exists between complicated strongyloidiasis and reduced host cellular immunity was first suggested nearly 50 years ago in independent studies that linked parasite dissemination to the occurrence of lymphoma [74] and corticosteroid therapy [75],[76]. Today, immunosuppression events, which often occur due to treatment with corticosteroids, are associated with an increased risk of disseminated disease [11]-[13],[73]. The reduction of cellular immunity is indeed a major cause of complicated strongyloidiasis, and experimental and natural hosts demonstrated that the production of Th2 cytokines and immunoglobulins is necessary to balance the host-parasite relationship and to eliminate intestinal nematodes [9],[22],[23],[73],[77]-[79]. The concept that treatment with corticosteroids favours hyperinfection and the dissemination of *S. stercoralis* in humans is based on the fact that although endogenous and exogenous steroids interfere with different immunoregulatory pathways during strongyloidiasis [14],[32],[80], these drugs affect the Th2 response more directly [81],[82], increasing the apoptosis of Th2 cells and damaging the function of eosinophils and mast cells [9],[27],[33]. Moreover, the production of IL-4 and IL-5 was inhibited more effectively than the production of IFN-gamma in T-cells by dexamethasone; this finding may account for the selective outgrowth of Th1-like T cells that was observed *in vivo* in patients treated with corticosteroids [82]. In addition to the host immune system, which may also be impaired due to iatrogenesis related to pharmacological immunosuppression, other potential determinants of severe strongyloidiasis that must be considered include conditions of reduced intestinal transit, co-infections, aspects related to hygiene and the nutritional state of the host and the presence of a high parasite burden associated with an active auto-infection process [83].

Data on the number of *S. stercoralis* females recovered from the intestines of marmosets during experimental complicated or uncomplicated strongyloidiasis and the evaluation of the developmental degree (i.e., juvenile or adult) and location (i.e., small intestine or large intestine) of the parasite females improved our understanding of the dynamics of parasitism. These data suggest a balance in the host-parasite relationship, with a virtually stable parasitic burden in immunocompetent hosts, because the number of nematodes that reach the small intestine is close to the number of nematodes that are recovered from the large intestine; this latter group might be easier for the host to expel. The loss of this balance may be related to the occurrence of complicated strongyloidiasis. Consistent with this hypothesis, in the small intestine of marmosets with *S. stercoralis* dissemination, in addition to the number, the ratio of juvenile females to adult females was significantly higher than that observed in primates that did not receive dexamethasone. Future studies are needed to determine whether these previously reported and/or additional unknown mechanisms are involved in the dissemination of *S. stercoralis* in marmosets.

Genta [34] stated that dysregulation is related to the dissemination of *S. stercoralis* and that the hypothesis of host immune control used to explain this phenomenon is vague because it ignores the possibility that the nematode may play a crucial role in its own regulation. The ability of the parasite to control its population size could be affected during the deregulatory process; thus, the intestinal numbers of *S. stercoralis* would grow indiscriminately until the parasite reached a critical value and killed the host. Although a direct effect of dexamethasone on the parasite cannot be ruled out, the data obtained in the present study indicate that another idea proposed by this same author (i.e., that corticosteroids administered to the host might spontaneously accelerate the transformation of rhabditiform larvae of *S. stercoralis* in the L3i due to structural similarities of the drug to natural parasite ecdysteroids [34]) is questionable. Because at the time of death of each *C. penicillata* with complicated strongyloidiasis, incontestable evidence indicated parasite dissemination and all drug had presumably been metabolized by the primate with enough time elapsed to allow the restoration of the host immune response, the idea that direct acceleration of the transformation of larvae of *S. stercoralis* by corticosteroids administered to the host is the main factor related to the occurrence of disseminated infection is not supported by the current results. In addition, infective larvae of the nematode were not usually observed in the faeces of dexamethasone-treated marmosets.

Thus, the occurrence of disseminated infection in marmosets did not occur solely due to effects of dexamethasone that were induced during the period of drug administration. Glucocorticoid administration to marmosets infected with *S. stercoralis* likely caused a sequence of events that resulted in inadequate control of parasite burden, providing a less inhospitable intra-host microenvironment for the parasite.

The mean percentage of *S. stercoralis* recovered from immunocompetent marmosets was higher than those previously reported for beagle dogs, whose mean recovery percentages of parasitic females were extremely variable [45], and jirds [36]; both of these models were experimentally infected with parasites of canine origin. However, the mean recovery rate of *S. stercoralis* in *C. penicillata* may be even higher because the lowest recovery ratio obtained for the animal P14 may have been because necropsy was performed at 12 DPI, when nematode larvae could still be migrating to the host tissues. In studies using *E. patas* infected with a human isolate of *S. stercoralis*[25]-[27], no information about the intensity of parasites was presented for uncomplicated infection, perhaps because these animals exhibited hyperinfection and dissemination of the parasite in the absence of immunosuppressive treatment, which precludes a proper analysis of the number of parasites. Schad *et al.*[39] previously stated that one of the advantages of the canine model, which was being proposed at the time, over the nonhuman primate host *E. patas* was the fact that complicated infection in this animal was not representative of human disseminated strongyloidiasis because the dissemination of *S. stercoralis* occurred very rapidly and independently of immunosuppressive treatment in the patas monkey. However, this potential limitation of nonhuman primate models was not observed in marmosets. In cases of hyperinfection and/or dissemination of *S. stercoralis* reported in *E. patas* and in dogs after glucocorticoid administration [26],[39],[40], as well as in *C. penicillata* in the current study, the number of larvae and adult worms recovered from animals with severe strongyloidiasis far exceeded the number of *S. stercoralis* L3i that was initially inoculated. In contrast, the increase in the number of parasitic females in the intestines of *M. unguiculatus* after glucocorticoid treatment was comparatively slight, and the number of recovered adult worms was even lower than the number of L3i used in the inoculum [36].

In human strongyloidiasis, the release pattern of larvae in the faeces oscillates widely in infected individuals and correct diagnosis often requires serial examinations [84],[85]. Autoinfection-related variations in the number of *S. stercoralis* females in the host were suggested as an explanation of this phenomenon [86]. In this study, similar findings were obtained and auto-infection occurred even during uncomplicated strongyloidiasis. However, the most substantial differences in the results of the qualitative and semi-quantitative parasitological tests were observed between dexamethasone-treated and untreated primates. The observation of higher numbers of females of *S. stercoralis* in the intestines of immunosuppressed hosts may reflect a non-static host-parasite relationship, in which constant attempts to achieve balance can result in increased or reduced fecundity of parasitic females in response to changes in the environment. Thus, the increased ratio of negative tests in the later patent period in immunocompetent marmosets may also be related to a reduction in the reproductive potential of *S. stercoralis* females, as sterile worms associated with ovarian degeneration and embryonation failure were common during chronic experimental strongyloidiasis in dogs [45].

In comparison to marmosets, the difficulties caused by the large size of patas monkeys and beagle dogs make the use of these animals to study experimental strongyloidiasis more difficult. However, it is important to note that the present study used a *S. stercoralis* strain isolated from humans. In addition, the origin of L3i (i.e., from cultures of human or *C. penicillata* faeces) was not related to marked changes in the evaluated parameters during strongyloidiasis in marmosets. In addition to the molecular differences established between *S. stercoralis* strains isolated from dogs and hominids [87] and the variable susceptibilities observed in epidemiological and experimental studies that used different strains and hosts [37],[38],[51],[88],[89], evidence indicates that biological differences exist between parasites of human and canine origin. The prepatency of infection with *S. stercoralis* of human and canine origin is different, even when the experimental infection is performed in the same host species [39],[46],[47],[88]. In *E. patas* and *C. penicillata* infected with human isolates the prepatent periods varied between 11 and 20 DPI [26] and between 13 and 22 DPI, respectively, and are consistent with the values accepted for human hosts [90]. Furthermore, the maintenance of *S. stercoralis* by successive passage among specimens of patas monkeys over four years did not alter the biology of the parasite, including the prepatent period [49]; this finding is similar to observations made in marmosets during the period in which the isolated strain of *S. stercoralis* was maintained (i.e., until the completion of the study) (data not shown). Just as the prepatency of human isolates of *S. stercoralis* failed to change significantly in the nonhuman primates *C. penicillata* and *E. patas*, no reduction of the prepatent period was detected after several successive passages of a strain of canine *S. stercoralis* canine in beagles [39],[49]. This finding suggests that parasites isolated from humans and dogs are able to better adapt to primates and canids, respectively.

Different isolates of *S. stercoralis* might also present peculiarities related to the larval migration route in the host. This observation provides an alternative explanation for the reduced importance attributed, in contradiction to the classical notion [37], to larval migration through the lungs and trachea in dogs infected with a strain of *S. stercoralis* that was isolated and maintained in beagle dogs [41],[44]. Despite substantial differences in the *S. stercoralis* migratory route, the methodologies used in these previous studies, which reached opposing conclusions, were well designed. In addition to findings related to the spatial and temporal dynamics of larval migration in canine hosts that were provided by compartmentalized mathematical models [41], the *in vivo* displacement of radioisotope-labeled L3i was monitored by scintography in ten-day-old beagles inoculated with the parasite, and a higher concentration of larvae occurred in the corresponding area of the abdomen in puppies, rarely in the thoracic region, between 3 and 6 DPI [44]. However, the high number of radiolabeled *S. stercoralis* L3i that was inoculated (i.e., 100,000/animal, subcutaneously) and the young age of the dogs, which prevented full development of the immune system, may have contributed these results. Despite convincing evidence, as previously discussed [34], the suggestion that the pulmonary route is not relevant could find resistance in the clinical setting, where *S. stercoralis* larvae are frequently observed in the bronchoalveolar lavage or sputum of patients with complicated strongyloidiasis [4],[6],[91]-[94]. Conversely, hundreds of parasite larvae were found in lumps of bloody mucus in the trachea of dogs submitted to tracheotomy before infection with *S. stercoralis*[37]. Although in this experiment, parasite passage to the host respiratory system was demonstrated and most larvae failed to become adults due to the physical interruption of the trachea, mild infections occurred and alternative possibilities for nematode development cannot be eliminated. Despite the absence of a specific experiment evaluating larval migration during the early phase of infection of marmosets with a human isolate of *S. stercoralis*, the lung histopathology of the specimen that died at 3 DPI revealed structures in the alveolar lumen that suggest that parasite passage to this organ occurred.

Histopathological findings in the lungs and intestines during both clinical forms of infection with *S. stercoralis* in marmosets were consistent with existing information for humans, and the injuries observed during disseminated strongyloidiasis were usually more severe and characterized by intense inflammation associated with erosions and/or ulcerations of the mucosa of the small (duodenojejunitis) and large (colitis) intestines, with focal or diffuse hemorrhagic processes in the lungs [83],[95]-[98]. Similar observations were reported during the natural infection of nonhuman primates [52],[55] and in experimental infections of patas monkeys [26] and dogs [40],[47],[99].

Data obtained related to the developmental stages of *S. stercoralis* from marmosets who died due to disseminated infection corroborate the idea that a deficiency in the mechanism of expulsion of adult worms associated with a virtually unlimited number of larvae generated by the auto-infection process occurs in patients with hyperinfection or disseminated strongyloidiasis [83]. Moreover, rhabditiform and filariform larvae and juvenile and adult females of *S. stercoralis* were found in extraintestinal sites, particularly in the stomach, lung and liver of marmosets that presented complicated infection. Although these findings, which constitute convincing evidence of disseminated strongyloidiasis, were previously reported in human and experimental infections [26],[39],[40],[47],[83],[100], they have not been discussed in detail. Despite conflicting information about the importance of pulmonary migration in primary infection with *S. stercoralis* in dogs [34],[37],[41],[44], the available experimental data related to the migration of parasite larvae through the host tissues in complicated strongyloidiasis exhibit greater convergence. In this clinical form, the parasite larvae migrating in the host tissues do not focus primarily on the pulmonary route. The results obtained in dogs indicate that during severe disease, only a small number of the total larvae are recovered from the lungs and/or trachea [39],[40]; this fact was corroborated in this study using marmosets. Thus, although larval migration during hyperinfection and disseminated infection was not the subject of the study performed by Schad *et al.*[41], their suggestion that during primary infection, the pulmonary route of migration is only one of many possible routes for the nematode to reach the host duodenum becomes even more relevant in the context of disseminated infection. Particularly with respect to complicated disease, the observation of *S. stercoralis* larvae, including larva currens [101]-[103], in other organs in the natural host and the isolation of various stages of the parasite from different organs of marmosets and a large amount of larvae from a fragment of skin and surrounding tissues from one specimen in this study support the idea that parasite larvae can migrate randomly, reaching a number of peripheral sites in the host.

In this study, in primates who died due to disseminated infection, juvenile and adult females of *S. stercoralis*, including some adult females with eggs in their uteri, were found in the usual ectopic sites, such as the stomach and large intestine, and in organs outside the digestive tube, particularly the lungs, trachea and liver. Similar findings were obtained during necropsies of dogs infected with this nematode in the absence [37],[63] or presence [40],[47] of pharmacological immunosuppression. An idea that was previously suggested [37],[63] and remained unpopular throughout most of the last century is useful in complementing our understanding of existing reports in the literature related to the observation of rhabditiform larvae of *S. stercoralis* in different host organs, including the lungs [39],[47],[96],[97],[100]. Fülleborn [37] did not mention the presence of all developmental stages of *S. stercoralis* in the lungs of experimentally infected dogs, but he suggested that adult females and rhabditiform larvae observed in the lungs could begin a new local cycle of the parasite inside the host (i.e., auto-infection). Later, new evidence from the same host species indicated that the progeny of parasitic adult females from the lungs are important for internal auto-infection [63]. Eggs, rhabditiform and filariform larvae observed in the bronchioles and other segments of the airway, oesophagus and stomach were considered to be products of adult worms located in the lung parenchyma or in the terminal segments of the tracheobronchial tree. The idea that at least one generation of *S. stercoralis* can occur in the host lungs was corroborated by the observation of adult parasites [84],[85],[104],[105] and eggs, which are sometimes embryonated, in the lungs and in the airways of human patients [4],[97],[98],[106]. Furthermore, the observation of 4 apparently fertile parasitic females of *S. stercoralis* together with 21 rhabditiform larvae/g of kidney tissue from an immunosuppressed dog infected with human nematodes [47] also indicates that the nematode may produce offspring in organs other than those of the digestive system. This finding was previously corroborated in immunosuppressed beagle dogs, which presented a large number of rhabditiform larvae and parasitic females in different organs [39]. Thus, although as a rule, the parasites likely do not reach maturity in the host lung and produce a new generation in that organ, this phenomenon does occur. Additionally, during complicated strongyloidiasis, adult females of *S. stercoralis* may give rise to new generations of the parasite in other organs of the host, beyond the intestines and lungs, on a small scale.

Finally, considering the high adaptive capacity of *S. stercoralis* during development, with the parasitic females reaching sexual maturity in adverse situations and even in ectopic sites, it seems plausible that during disseminated infection, as an alternative, some larvae could develop completely in the host intestines without migrating through other organs for parasite multiplication within the host. In fact, the finding that juvenile females of *S. stercoralis* (on average, approximately 1/3 of the parasitic forms) are present in the large intestines during complicated strongyloidiasis in marmosets suggests that the parasite cycle may occur locally. Additional evidence for this notion was provided by the finding that all of the developmental stages of the parasitic life cycle were present in both the small and large intestines of immunosuppressed hosts, as already mentioned.

The obligatory passage of *S. stercoralis* larvae through the lungs, where the nematode would receive stimuli for its growth has been questioned in the canine model, as previously discussed [41],[44]. Evidence that the entire development of some larvae of *S. stercoralis* occurred in the intestines of marmosets during disseminated strongyloidiasis was presented in this study. A portion of the filariform larvae and juvenile parasitic females that were observed in the small intestines of marmosets may have originated from the lung via the trachea, larynx, pharynx, esophagus and stomach. However, one should also consider that there was an unusually high proportion of juvenile females in the large intestines of *C. penicillata* and that these observations regarding the degree of development of the intestinal parasitic females of *S. stercoralis*, in combination with the low numbers of larvae obtained from the lungs of these primates and the presence of large numbers of larvae in a skin specimen, also indicate that large-scale orderly pulmonary migration does not occur during complicated experimental strongyloidiasis.

## Conclusions

The data obtained in this study illustrated that *C. penicillata* is a susceptible model for infection with *S. stercoralis* of human origin. Both the complicated and uncomplicated forms of strongyloidiasis that are observed in the natural host can be reproduced in these nonhuman primates, making this model useful for the investigation of various aspects of this infection, such as immunopathology, drug assays and/or the development of new prophylactic measures. Successful consecutive passages of a human strain of *S. stercoralis* in marmosets indicate that laboratory maintenance of this organism is possible, at least long enough to perform the proposed biomedical experiments. Moreover, as long as the ethical principles were in compliance, the achievement of a substantial number of larvae and adult females of nematodes in these animals is relevant for the production of specific antigens for scientific and diagnostic purposes. In parallel, marmosets infected with *S. stercoralis* facilitated the collection of additional information related to the basic biology of the parasite, providing new insights into the autoinfective cycle, particularly during disseminated infection. In addition to the comments on the imbalance in the host-parasite relationship induced by corticosteroids from a parasitological perspective, the other two main points related to the biology of *S. stercoralis* addressed in this study are the indication that a direct effect of dexamethasone on the parasite is not required for its dissemination in the nonhuman primate host and confirmation that during disseminated infection the full development of *S. stercoralis* can occur in the host intestines and other organs.

## Authors’ original submitted files for images

Below are the links to the authors’ original submitted files for images. Authors’ original file for figure 1 Authors’ original file for figure 2 Authors’ original file for figure 3 Authors’ original file for figure 4
