# Supplementary figures and images for: Strongyloides stercoralis infection in marmosets: replication of complicated and uncomplicated human disease and parasite biology
Source: Parasit Vectors. 2014 Dec 12;7:579. doi: 10.1186/s13071-014-0579-2 (PMC4287166; doi:10.1186/s13071-014-0579-2)

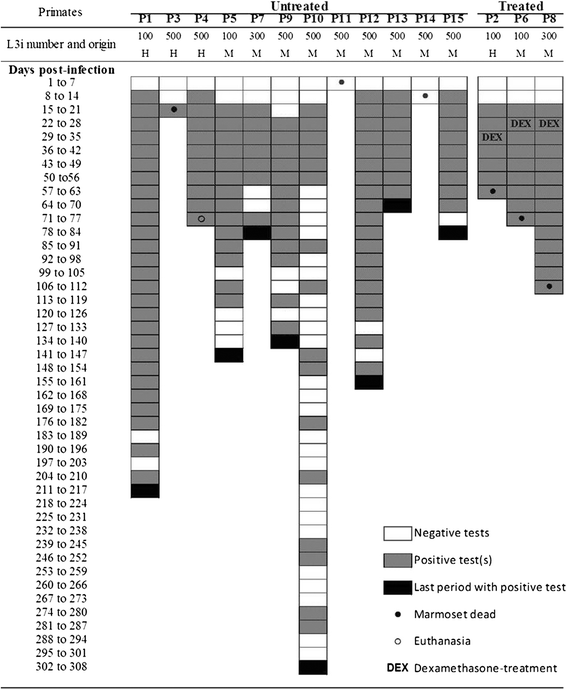

Supplement: Supplementary file 1 — Authors’ original file for figure 1 [file 13071_2014_579_MOESM1_ESM.gif]

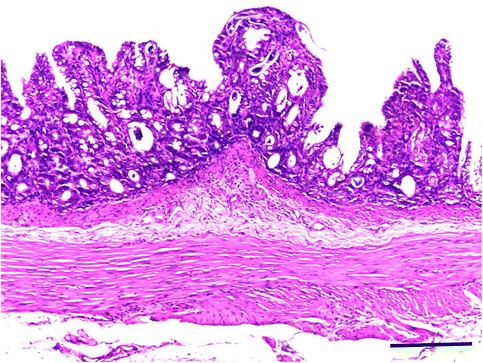

Supplement: Supplementary file 2 — Authors’ original file for figure 2 [file 13071_2014_579_MOESM2_ESM.gif]

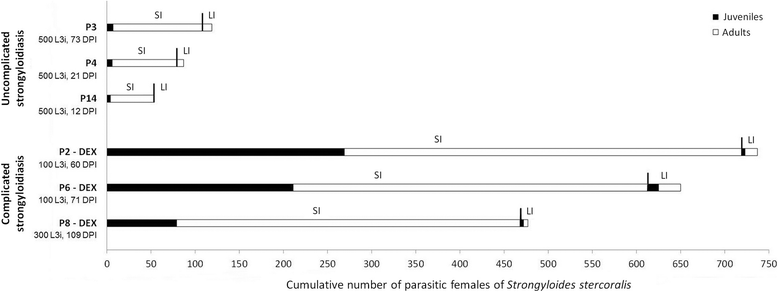

Supplement: Supplementary file 3 — Authors’ original file for figure 3 [file 13071_2014_579_MOESM3_ESM.gif]

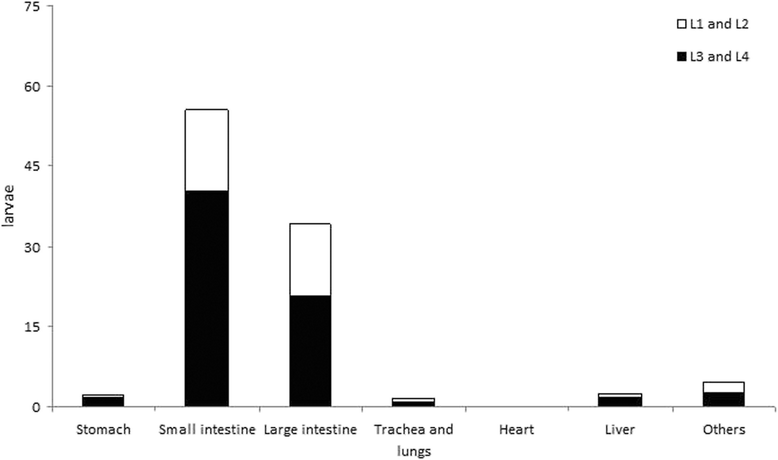

Supplement: Supplementary file 4 — Authors’ original file for figure 4 [file 13071_2014_579_MOESM4_ESM.gif]
